# Addressing dermatologic concerns and teledermatology in undomiciled and sheltered populations

**DOI:** 10.1007/s00403-024-03274-9

**Published:** 2024-08-19

**Authors:** Kennedy Gallagher, Sahithi Talasila, Anna Bistline, Rebecca Krain, Leena Ramani, Elizabeth Jones

**Affiliations:** Department of Dermatology and Cutaneous Biology, Thomas Jefferson University, 233 S. 10th Street, Philadelphia, PA 19107 USA

**Keywords:** Homelessness, Dermatologic disease, Teledermatology, Healthcare disparities, Undomiciled populations, Access to care

## Abstract

Homelessness in the United States is a significant public health issue, with dermatologic disease being the most prevalent health concern among the undomiciled and sheltered populations. Despite a growing need for dermatologic care, the supply of dermatologists remains insufficient, contributing to disparities in healthcare access for this vulnerable group. This review aims to detail the spectrum of dermatologic conditions experienced by homeless individuals, identify barriers to adequate care, and explore teledermatology as a potential solution to bridge these gaps. A comprehensive literature review was conducted, analyzing studies and reports on dermatologic issues prevalent among the homeless population and the efficacy of teledermatology in addressing these concerns. Homeless individuals face a wide range of dermatologic problems, from common conditions like acne and eczema to severe issues such as cellulitis, leg ulcers, and skin cancer. Drug abuse, domestic and sexual abuse, and parasitic infestations further complicate the dermatologic health of this population. Teledermatology has emerged as a promising tool to enhance access to dermatologic care, showing significant improvements in clinical outcomes and accessibility, especially in underserved urban settings. However, challenges remain, such as the digital divide affecting the elderly and low-income populations, which could potentially exacerbate disparities. Addressing the dermatologic needs of the homeless population requires a multifaceted approach. Teledermatology offers a viable solution to improve care access and efficiency, but additional efforts are necessary to ensure inclusivity and avoid further marginalization. Volunteer-driven multidisciplinary clinics also play a crucial role in providing care, though they face challenges in continuity and resource availability. Future strategies should focus on integrating teledermatology with other supportive services to create a comprehensive care model for this underserved population.

## Introduction

Homelessness is considered a major public health crisis in the United States (U.S.). Dermatologic disease is the most common health problem among the homeless [1]. With homelessness rates displaying an overall 6% increase since 2017 and counts of individuals (421,392) and chronically homeless individuals (127,768) reaching record highs in 2022, it is important to approach the dermatologic care of these patients with a closer eye [2]. Additionally, it has been demonstrated that access to and supply of dermatologists is limited. The recommended density for adequate dermatologic care is 4.0 per 100,000 people [3]. Although dermatologist density has increased from an estimated 1.9 per 100,000 individuals in 1970 to 3.4 in 2017, the recommended density for adequate dermatologic care has​ not been met [3, 4]. This combination of overwhelming demand for dermatological care alongside a glaring undersupply of dermatologists has contributed to a disparity in access to care, leaving a large portion of health problems within the homeless population unaddressed. This review essay will detail the spectrum of dermatologic conditions experienced by the homeless population and the various problems that hinder adequate access to care, as well as explore teledermatology as a solution to bridging such gaps. Implementing a better methodology for access to skin care is essential in meeting the unmet needs of the homeless population.

## Review

### Problems of neglect

Dermatologic disease among the homeless is a common problem that often goes neglected until conditions become severe and debilitating. In one study, admissions to an inpatient dermatology unit over a 3 month period showed that 46% of admitted patients were either homeless or lived in a shelter, 48% had skin infections including cellulitis, and 81% of patients admitted for cellulitis or pyodermas were homeless [5]. The undomiciled and sheltered populations experience a wide range of skin conditions from common dermatologic problems like acne, psoriasis, seborrheic dermatitis, and pruritic conditions like prurigo nodularis and atopic dermatitis to infestations of scabies and pediculosis, as well as more unique problems attributable to street living like leg ulcers, immersion foot, and frostbite [6].

In people experiencing homelessness (PEH), foot-related problems continue to be a major cause of morbidity and mortality that often go overlooked. A systematic review found that up to two thirds of homeless individuals reported a foot-related health concern [7]. Prolonged standing, limited access to clean socks and properly fitting footwear, and lack of hygiene lead to common presenting concerns amongst the homeless population including corns and calluses, toenail onychomycosis, and pitted keratolysis. Lack of or unstable housing and exposure to natural elements promotes frostbite injury from prolonged cold as well as immersion or trench foot from prolonged moisture. Foot injuries including deformities, trauma, and fractures are also common [8]. Poorly controlled diabetes within this population frequently predisposes individuals to development of various foot pathologies. A study screening homeless shelter residents for diabetes found that 41% of homeless individuals with diabetes had difficulty walking, 42% had a loss of foot sensitivity, 43% had permanently reduced mobility, and 17% previously had lower limb amputation [9]. Other comorbidities including alcohol-induced peripheral neuropathy, peripheral vascular disease, and vasoconstrictive drug dependence can contribute to poor wound healing and increased infection risk. Additional foot afflictions such as lymphedema, venous stasis dermatitis, and tinea pedis can be predisposing factors for cellulitis, ulcers, chronic wounds, osteomyelitis, gangrene, and squamous cell carcinoma [5, 7].

An unfortunate reality for many shelter-living individuals who seek out care for dermatologic problems is that they often must hide their affliction for fear of ostracism by other residents who assume an infectious etiology. These individuals may not have a place to bathe, tend to their wounds, or apply medication. An extreme example of this neglect to care for dermatologic conditions is the ability to prevent progression of psoriasis to the life-threatening complication of psoriatic erythroderma when given access to topical therapy and an appropriate setting for treatment application [5]. Another reality for homeless patients once discharged from the hospital is their inability to recuperate appropriately. Most patients in this demographic rely on emergency departments for health care and are likely to deteriorate, requiring re-hospitalization for the same problem [5].

A long-term consequence of neglect within this group of individuals is that of skin cancer. Prolonged sun exposure, limited education regarding skin cancer risks, suboptimal sun protective measures, and reduced access to professional screening contribute to increased rates of malignant and premalignant growth among PEH compared to the general population (25%, 6.1%, *P* < .001) [8]. Studies have shown that 50–79% of PEH have never used sunscreen and 71–76% of PEH have never had a screening skin exam [10]. Lack of preventive measures including appropriate education, affordable access to protection, and screening efforts make the homeless population at greater risk for skin cancer.

### Problems from drug abuse

In recent years, there has been a decline in drug usage rates, particularly casual drug abuse, although alcohol and cocaine use continue to rise [5]. Current drug usage trends among homeless individuals demonstrate the complex interplay between substance abuse and homelessness, with various factors influencing drug use patterns in this vulnerable group. Studies have shown that homeless individuals have a significantly higher prevalence of substance abuse or dependence, with approximately two-thirds of them having a history of substance abuse [11]. Substance use disorders, including alcohol, opioids, methamphetamine, and cocaine use, are prevalent among the homeless population, often serving as coping mechanisms for the challenges associated with homelessness and concurrent mental health issues. The experience of homelessness itself contributes to drug use trends due to factors such as lack of stable housing, exposure to violence, and limited access to resources, which can increase the likelihood of substance abuse as a form of self-medication or escape. Homeless individuals with a history of substance use also report higher levels of trauma, mental health problems, and chronic health conditions [5]. Additionally, the availability and accessibility of drugs play a significant role in drug usage trends among the homeless population, as these individuals often face higher exposure to drug markets due to their living conditions and limited resources, making them more susceptible to drug initiation and relapse [5].

An increasing concern is the HIV epidemic among intravenous drug abusers (IVDAs), their partners, and their children. It has been estimated that around 300,000 IVDAs nationwide may be infected with HIV, and 8% of new IVDAs become infected each year [5]. These environments are often marked by violence, involving drug dealing, possession, robbery, and prostitution, which can lead to injuries such as trauma, gunshot wounds, knife wounds, and beatings. Furthermore, certain drugs, such as cocaine and phencyclidine (PCP), can contribute to increased suspicion and violent behavior.

In individuals with drug abuse, the skin can exhibit various clues that indicate drug use, such as burns, frostbite, gangrene, bullae, scars, hyperpigmentation consistent with track marks, ecchymoses, cellulitis, punched-out atrophic lesions from skin picking or drug injection, and granulomas caused by additives in street drugs [5]. Hiding scars and track marks through tattoos is a common method employed by drug users. However, the most common skin symptom of drug abuse is infection. Reusing needles can lead to complications such as abscesses, cellulitis, gangrene, intra-arterial vasospasm, and skin necrosis, which may necessitate amputation [5].

Cocaine use presents with specific dermatological findings, including nasal septum perforation, mucosal erythema, and madarosis (loss of eyebrows and eyelashes) due to exposure to hot vapors from smoking this substance. Another common phenomenon associated with cocaine use is self-mutilation and skin picking, often referred to as the “cocaine crawlies,” which leads to pruritic sensations in many users. Interestingly, cocaine use can heighten sexual interest while causing difficulties in achieving orgasm, which has been identified as one of the contributing factors to the resurgence of syphilis in certain populations, as individuals may resort to engaging in risky behaviors to obtain drugs [5].

Intravenous drug abuse, particularly with substances such as heroin and morphine, is associated with characteristic symptoms such as pseudo-acanthosis nigricans, thrombophlebitis, cheilitis, vasculitis, and reports of drug eruptions, systemic amyloidosis, thrombosis, and necrosis [5]. Additionally, the use of fentanyl has been on the rise in the Philadelphia population. Within this illicit drug supply, the presence of a veterinary anesthetic called Xylazine has been increasing as an adulterant and has been implicated as the source of many overdose deaths [13]. Xylazine produces a unique drug-associated skin ulcer. These wounds typically manifest as dry dead tissue (eschar), often presenting as purulent, foul-smelling lesions that occur in sites distal to the injection site. Local hospitals have reported an increase in skin and soft tissue infections associated with xylazine use, with some cases progressing to sepsis. The mechanism of action behind these skin ulcers is believed to involve direct vasoconstriction of nearby blood vessels, leading to reduced blood flow and oxygen supply to the skin. Prolonged use of xylazine exacerbates vasoconstriction and the resulting oxygenation deficit, ultimately leading to the development of skin ulceration or severe soft tissue infections in the form of abscesses or cellulitis. The compromised blood flow further hampers wound healing and increases the likelihood of infection [14].

### Problems from domestic and sexual abuse

Domestic and sexual abuse are significant issues that affect individuals across various demographics, contributing to physical and emotional trauma. These forms of abuse are not only detrimental to the immediate health and well-being of victims but are also recognized as common causes of homelessness. Many individuals who experience domestic violence are forced to leave their homes to escape abuse, resulting in a significant portion of the homeless population having histories of such trauma.

One significant predictor of sexual and physical abuse among the pediatric population is the prevalence of families living below the poverty line. In impoverished communities, child abuse is reported at a rate of 2–3 cases per 1000 children, while in homeless populations, the rate increases to 8.8 cases per 1000 children [5]. Several risk factors have been identified in relation to child abuse, including the mental and psychological health of parents, life stressors, unemployment, drug abuse, medical problems in the child, and the presence or absence of familial support systems. Disturbingly, the abuser is often someone known to the child [5]. Identifying and reporting cases of child abuse requires the expertise of experienced healthcare professionals who can then communicate the findings to the appropriate agency.

Abused children often exhibit skin findings that include injuries in various stages of healing, unusual patterns of scars, and keloids. These injuries and indicators of abuse are frequently a response to the child’s behavior and can be caused by a variety of objects such as belts, paddles, and sticks. Physical assault can take the form of kicking, punching, slapping, and choking, resulting in bruises, lacerations, scratches, as well as fractures and other traumatic injuries. Deliberate burns and disfigurement may be inflicted using scalding bathwater, stovetops, hairdryers, or cigarettes. Despite outward appearances of being well-groomed and cared for, neglected toddlers may exhibit signs of malnourishment. Dermatitis neglecta, also known as unwashed dermatosis, results from inadequate hygiene and manifests as hyperpigmented, verrucous plaques due to the accumulation of sweat, sebum, and debris on the skin. Management involves regular cleansing and improved access to hygiene facilities [2]. It is essential to consider certain dermatologic conditions that mimic trauma, including bullous diseases, lichen sclerosus, Ehlers-Danlos syndrome, and staphylococcal scalded skin syndrome. Before reporting an incidence, it is also important to consider cultural practices such as cupping [5].

The signs of domestic abuse often leave emotional scars alongside physical injuries. Patients may primarily present with psychosomatic complaints, which are the primary concerns voiced during clinic consultations. Sexual abuse signs can include similar manifestations as previously mentioned, along with the presence of sexually transmitted diseases (STDs). Anal mucosal injuries are often observed in male victims, and unfortunately, the perpetrator is typically someone known to the victim [5]. Signs of neglect and abuse in the elderly population mirror those seen in children. Additional findings may include ecchymoses, urine burns, decubitus ulcers, freeze injuries, and poor hygiene. Poor nutrition and weight loss should also raise concerns [5].

### Problems from parasites

Various parasites, such as certain mites, fleas, ticks, and worms, can infest the skin and cause significant discomfort and damage. These infections can lead to symptoms such as itching, redness, inflammation, and the formation of skin lesions. In some cases, the parasites may burrow into the skin, causing tunnels or tracks. Others may bite or feed on the skin, leading to localized irritation or allergic reactions. The presence of parasites on or within the skin can disrupt its normal function and integrity, potentially resulting in secondary infections, scarring, or even systemic complications.

In a study conducted in Paris, France, the estimated prevalence of scabies among individuals sleeping in public places was 6.5%, while the prevalence of pediculosis corporis was 5.4% [9]. It is worth noting that the incidence of scabies and pediculosis corporis among those in shelters was significantly lower at 0.4% and 0.15%, respectively [9]. Multivariable analysis revealed that some of the risk factors associated with scabies and body lice infestation in public places included being a woman, squatting in a building, and not possessing a sleeping bag. Additional potential risks identified in the study were begging, a history of pubic lice infestation, and refusal to use communal showers [9]. Another study reported that scabies accounted for 56.5% of consultations for homeless individuals in Paris and was found in 58% of infectious disease and dermatologic consultations among migrants arriving in Italy [11]. However, in lower-income countries, scabies is more prevalent among children due to factors such as overcrowding, bed-sharing, and under-recognition of the disease [11]. The typical presentation of scabies involves intensive nocturnal itching, with symptoms developing 4–6 weeks after initial exposure, although reinfection may become noticeable after a few days.

Infections with scabies can have significant implications, including secondary bacterial infections caused by Staphylococcus or Streptococcus species resulting in high risk of mortality from secondary sepsis. Less severe complications may include abscesses, cellulitis, and soft-tissue necrosis [11]. Similarly, pediculosis (lice infestation) shares many risk factors with scabies. Clinical findings of body lice infestation include itching and common skin lesions such as linear excoriations, post-inflammatory hypopigmentation, and lichenification. These are typically found along the neck, shoulders, back, flank, and waist, which correspond to sites of contact between clothing and the skin [10].

### Teledermatology to address care gaps

The overwhelming lack of dermatologic services and care for the undomiciled and sheltered population has demanded strategic solutioning from within healthcare systems. The combination of disproportionately increased risk and exposure to certain skin-related conditions, along with lack of access to appropriate dermatologic care, makes this extremely vulnerable population prone to long-term complications. Additionally, the shortage of dermatologists in the U.S. has contributed to longer wait-times for office-based appointments, and is especially problematic for the uninsured, Medicaid, and rural populations [15]. Several approaches have been explored to address this care gap, the main method being implementation of teledermatology. Teledermatology is conducted in different forms including real-time video or telephone conferencing, asynchronous store-and-forward (SAF) teledermatology utilizing transfer of digital images, and a hybrid model merging live video with digital photos.

Recent findings have shown teledermatology to be a cost- and time-effective tool that positively impacts clinical outcomes and increases accessibility and speed of care for medically underserved populations. In a study evaluating the impact of SAF teledermatology delivery on outpatients in underserved primary care clinics in Philadelphia, PA, the management and diagnostic concordance between dermatologists and primary care providers was only 23% and 22% for skin conditions, respectively [3]. This high rate of discordance is promising for enhanced clinical outcomes with the introduction of dermatology-specific care [3]. Additionally, provider-to-provider teledermatology and consult services allow for beneficial exchange of knowledge with other providers. A recent study implementing a targeted educational program for primary care providers reported significant improvement in diagnostic and management confidence and concordance of dermatologic skin condition diagnoses [22]. Notably, it was also reported that at least 61% of consults from the Philadelphia-based clinics would not otherwise have received dermatology input, and 77% of consults were adequately managed with teledermatology alone [3]. Not only does this demonstrate an overwhelming extension of access to services through teledermatology, but it also promotes greater care efficiency and provider productivity. Such efficiency and productivity of in-person dermatology appointments is hindered by a clinic nonattendance rate that is highly correlated with insurance status, with uninsured patients and Medicaid recipients most likely to miss their appointments [16]. Recently, a study comparing pediatric in-person and telemedicine no-show rates from 3 safety-net dermatology clinics in New York City were significantly and similarly reduced from 47%, 47%, and 45–26%, 37%, and 36% (*p* < .001 at all sites), demonstrating an appreciable benefit from the increased accessibility and convenience afforded by telemedicine [17–20].

Despite the seemingly advantageous impact of teledermatology on underserved patient populations, it is important to explore the possibility that this relatively new method of care delivery could lead to further exclusion and disparity. Interestingly, the use of telemedicine at the 3 safety-net dermatology clinics in New York City had a very different impact on patients > 65 years old, showing an increase in telemedicine nonattendance rates when compared to in-person at 2 of the 3 sites from 31%, 29%, and 35–31%, 31%, and 51% (*p* < .001, *p* = .49) [16, 21, 22]. Susceptible populations such as low-income, undomiciled, and Medicare patients > 85 years old may lack the high-speed internet, digital technology resources, and knowledge to support access to and use of such services [23]. A telecare pilot service study based out of Budapest, Hungary, incorporated on-site assistants within participating homeless shelters to facilitate telecare appointments and support technological troubleshooting. On the 5-point Likert scale, participants expressed that the on-site assistance was helpful for them (mean = 4.68, SD = 0.72), likely contributing to resultant high attendance and satisfaction scoring [24]. Additional efforts such as this must be taken to ensure that teledermatology as a solution to close care gaps does not cause a widening digital divide and create an even more disparate system than was present before [25].

Another method used to address the dermatologic needs of the undomiciled population of Philadelphia is the use of volunteer-dermatologists, resident-physicians, and medical students to run multidisciplinary clinics to provide specialty care to the uninsured and underserved population of Philadelphia. Several of these clinics exist across the city, such as the dermatology clinic at Puentes de Salud run by University of Pennsylvania, and the ACTS Clinic run by Thomas Jefferson University [4]. The community health clinic at University of Pennsylvania implemented a teletriage system that shortened wait times and allocated in-person appointments based on acuity and complexity of the presenting problem. The ability to manage certain conditions with teledermatology alone increased in-person appointment availability by 18%, which is an average of 1.4 of 8 appointments per month [4]. A significant challenge with this type of volunteerism approach is the lack of care continuity. The physicians who staff this clinic change from month-to-month, and this clinic is typically only staffed once per month as opposed to the primary clinic which is staffed once a week. An alternative to clinic-based and teledermatologic care is “Street Dermatology.” This clinical model has been trialed specifically to cater to people experiencing unsheltered homelessness by bringing dermatology services directly to the streets, essentially eliminating the need for formal health care visits. The volunteer street medicine providers involved in executing this engagement approach were successful in making a total of 125 dermatologic diagnoses over a 6-month period, and providing both education and treatments related to specific conditions. As with any of the models mentioned, there is inherent unpredictability to follow-up and continuation of care [26]. There is not a perfect solution to help ameliorate the barriers to dermatologic care that this population faces, however despite certain drawbacks, it appears that teledermatology is a critical step in the right direction.

### Factors to follow-up adherence

A recent article published in JAAD looked at the factors limiting adherence to follow-up dermatology appointments in the homeless population in Salt Lake City, Utah. Mental health diagnoses were found to pose a significant limiting factor when it came to likelihood of follow-up appointments as well as care compliance. National averages of mental health burden being at 18.5% of the general U.S. population and 46% of the homeless U.S. adults warrant thorough investigation to identify these high-risk patient populations for potential non-adherence [12]. Further unique obstacles that these individuals have to face, such as language barriers, lack of insurance, transient address or telephone contacts, unreliable accessibility to transportation and fear of stigmatization contribute to low follow-up adherence [25, 27, 28].

## Conclusion

With homelessness being a major health problem in the U.S. and skin disease being extremely prevalent among homeless individuals, it is important to understand this population’s specific clinical needs and barriers to accessing care. The spectrum of skin issues in the typical underserved population includes problems from neglect, drug abuse, domestic and sexual abuse, and parasitosis. Many models for intervention have been proposed and trialed, with teledermatology representing a promising avenue for dermatology-related education, prevention, diagnoses, and treatments. Additional teledermatology solutioning as well as alternative methods of care delivery continue to be evaluated to address this gap in care within an underserved undomiciled and homeless population.

## Data Availability

No datasets were generated or analysed during the current study.
